# Simvastatin Inhibits Endotoxin-Induced Apoptosis in Liver and Spleen Through Up-Regulation of Survivin/NF-κB/p65 Expression

**DOI:** 10.3389/fphar.2019.00054

**Published:** 2019-02-15

**Authors:** Lana Nežić, Ljiljana Amidžić, Ranko Škrbić, Radoslav Gajanin, Eugenie Nepovimova, Martin Vališ, Kamil Kuča, Vesna Jaćević

**Affiliations:** ^1^Department of Pharmacology, Toxicology and Clinical Pharmacology, Faculty of Medicine, University of Banja Luka, Banja Luka, Bosnia and Herzegovina; ^2^Institute of Pathology, University Clinical Center of Republic of Srpska, Faculty of Medicine, University of Banja Luka, Banja Luka, Bosnia and Herzegovina; ^3^Department of Chemistry, Faculty of Science, University of Hradec Králové, Hradec Králové, Czechia; ^4^Department of Neurology, Charles University in Prague, Faculty of Medicine in Hradec Kralove and University Hospital, Hradec Kralove, Czechia; ^5^Malaysia-Japan International Institute of Technology, Universiti Teknologi Malaysia, Kuala Lumpur, Malaysia; ^6^Department of Experimental Toxicology and Pharmacology, National Poison Control Center, Military Medical Academy, Belgrade, Serbia; ^7^Medical Faculty of the Military Medical Academy, University of Defense in Belgrade, Belgrade, Serbia

**Keywords:** simvastatin, endotoxin, hepatocytes, lymphocytes, apoptosis, survivin, NF-κB/p65

## Abstract

Endotoxemia is associated by dysregulated apoptosis of immune and non-immune cells. We investigated whether simvastatin has anti-apoptotic effects, and induces hepatocytes and lymphocytes survival signaling in endotoxin-induced liver and spleen injuries. Wistar rats were divided into the groups pretreated with simvastatin (20 or 40 mg/kg, orally) prior to a non-lethal dose of lipopolysaccharide (LPS), the LPS group, and the control. The severity of tissue inflammatory injuries was expressed as hepatic damage scores (HDS) and spleen damage scores (SDS), respectively. The apoptotic cell was detected by TUNEL (Terminal deoxynucleotidyl transferase dUTP Nick End Labeling) and immunohistochemical staining (expression of cleaved caspase-3, and anti-apoptotic Bcl-xL, survivin and NF-κB/p65). Simvastatin dose-dependently abolished HDS and SDS induced by LPS (*p* < 0.01), respectively. Simvastatin 40 mg/kg significantly decreased apoptotic index and caspase-3 cleavage in hepatocytes and lymphocytes (*p* < 0.01 vs. LPS group, respectively), while Bcl-XL markedly increased accordingly with simvastatin doses. In the simvastatin, groups were determined markedly increased cytoplasmic expression of survivin associated with nuclear positivity of NF-κB, in both hepatocytes and lymphocytes (*p* < 0.01 vs. LPS group). Cell-protective effects of simvastatin against LPS seemed to be mediated by up-regulation of survivin, which leads to reduced caspase-3 activation and inhibition of hepatocytes and lymphocytes apoptosis.

## Introduction

Sepsis is defined as infection-induced multiple organ dysfunctions (MODS) associated with initial pro-inflammatory response, followed by dysregulation of host response to infection (Vincent et al., 2013; Singer et al., 2016). Current data indicated that sepsis-induced organ failure and cell-death are results of necrosis, apoptosis and autophagy (Choi et al., 2013; Hotchkiss et al., 2013; Oami et al., 2018). Apoptosis presents an important mechanism for cell death in sepsis, and affects non-immune (Smeding et al., 2012; Koçkara and Kayataş, 2013; Yan et al., 2014) and immune cells (Wang, 2015) through both death receptor- and mitochondrial-mediated pathways (Chang et al., 2007). Dysregulated apoptosis is proposed to contribute to organ injuries, but also lead to immunosuppression due to loss of immune cells (Hotchkiss et al., 2013). Considering the fact that controlling of pro-inflammatory cytokines has not improved clinical outcomes in clinical trials, a promising strategy in sepsis treatment might be focused on cell survival (Cohen et al., 2012; Marshall, 2014).

Pathogen-derived substance with immunological properties, such as lipopolysaccharide (LPS), the component of Gram-negative bacteria cell wall, has been shown to induce sepsis-like conditions *in vivo*. LPS induces over-production of pro-inflammatory cytokines, such as tumor necrosis factor-alpha (TNF-α) and interleukin-6 (IL-6) that cause inflammatory tissue injuries and apoptosis (Smeding et al., 2012; Hotchkiss et al., 2013; Koçkara and Kayataş, 2013; Yan et al., 2014). Shinozaki et al. (2010) demonstrated that inhibition of pro-apoptotic molecules caspase-3 and Bax and Bim expression attenuated LPS-induced liver and spleen dysfunction and reduced mortality. Furthermore, it has been shown that caspase inhibitors and death receptor blockers prevent lymphocyte apoptosis and improves survival in sepsis (Hattori et al., 2010). TNF-α can induce apoptosis in various cells but also activate specific intracellular survival pathways through NF-κB activation. Nuclear factor-kappa B (NF-κB) has an important role in inflammation and immune regulation, but also its activation initiates intracellular survival pathway through up-regulation of anti-apoptotic Bcl-2, Bcl-Xl, and inhibitors of apoptosis (IAP) expression, such as survivin (Hotchkiss et al., 2013; Wang, 2015). Survivin is an oncoprotein with a dual cellular function that depends on subcellular localization, in which its nuclear expression promotes cell mitosis; whereas survivin in the cytosol exerts anti-apoptotic activity by down-regulation of caspase-3 activation or binding to pro-apoptotic protein Smac/Diablo (Altieri, 2010). Accumulated evidence has shown that survivin exerts cell-protection in non-malignant conditions, i.e., in immune cells (Andersson et al., 2015), cardiomyocytes (Lee et al., 2015; Bo et al., 2017), small intestine (Scheer et al., 2017), and hepatocytes (Wang et al., 2010). However, whether the signaling pathway survivin/NF-κB plays a role in LPS-induced inflammatory tissue injury remains to be elucidated.

Our previous experimental studies have demonstrated that simvastatin, a lipid-lowering drug, exerts anti-inflammatory properties by decreasing skin inflammatory injury (Nežić et al., 2009a) or production of TNF-α, IL-1β, and IL-6 in acute systemic inflammation (Nežić et al., 2009b). Several studies in experimental model of sepsis have also shown that simvastatin improved survival, attenuated activation of stress-activated protein kinase JNK/SAPK and apoptosis in liver and spleen (Shinozaki et al., 2010), reduced cardiomyocyte apoptosis through decreased p53 expression (Buerke et al., 2008), and mitigated lung tissue injury by inhibiting toll-like receptor 4 (TLR4)/NF-κB signaling pathway and expression of caspase-3 and Bax (Wang et al., 2018).

In an effort to understand the mechanisms through which simvastatin may express cell-protection against LPS-induced apoptosis, we analyzed its protective effects on liver and spleen inflammatory injury and extent of apoptosis, and then its capability to up-regulate expression of the survival signaling survivin/NF-κB pathway.

## Materials and Methods

### Experimental Animals

In this experiments male Wistar rats, 6–8 weeks old (200 to 220 g) bred at the Department for Experimental Animals, Military Medical Academy, Belgrade, Serbia were used. All rats were housed in plastic cages (*n* = 5) (Macrolon^®^ cage type 4, Bioscape, Germany) with certificated sawdust bedding (Versele-Laga, Deinze, Belgium) under controlled environmental condition (temperature of 22 ± 2°C, relative humidity of 55 ± 15%, 15–20 air changes/h, and 12 h light/dark cycle). During the whole study period, animals had free access to standard laboratory food (Veterinary Institue Subotica, Serbia) and fresh water *ad libitum*. In addition all the experimental procedures and environmental conditions were adopted by the Ethics Committee for Experiments on Animals, Military Medical Academy, Belgrade, Serbia (approved study protocol no.: 282-12/2002), and National Guideline for Animal Welfare, Republic of Serbia (decision No. 323-07-04943/2014-05/1).

### Drugs

Drug simvastatin (produced and kindly donated by Krka, Novo Mesto, Slovenia) was dissolved in 0.5% methylcellulose (Sigma, Taufkirchen, Germany), as 10 or 20 mg/ml stocks. Endotoxin (LPS) from Escherichia coli serotype 0127:B8 (Sigma Aldrich, Munich, Germany) was administered intraperitoneally (ip) immediately after dilution with sterile pyrogen-free physiologic saline. All invasive procedures in animals were conducted under aseptic conditions.

### Experimental Design

We used simvastatin in the previously examined doses (20 or 40 mg/kg per os) that were shown as the protective against the single median lethal dose (LD_50_) of 22, 15 mg/kg ip of LPS in rats (95% CI 16.5–29.1) (Nežić et al., 2009b). The selected dose of simvastatin were comparable to those that had previously been used in rat/murine studies *in vivo* (typically 10–100 mg/kg/day), and were higher than those used in humans because of a significant up-regulation (3- to 8-fold) of HMG-CoA reductase induced by statin treatment in rodents (Nežić et al., 2009b; Liu et al., 2012; Morel et al., 2017). In our experimental model of sepsis we challenged the animals with a non-lethal single dose of LPS ip (0.25 LD_50_/kg), a confirmed model that induces the strongest inflammatory effects of the all animal models for acute systemic inflammation, including immune cell infiltration, oxidative stress and apoptosis of organ tissues (Nežić et al., 2009b; Seemann et al., 2017). Animals were randomly divided into four experimental groups (*n* = 6 rats per group). The animals were treated as follows: (1) Control (0.5% methylcellulose 1 ml/kg ip), (2) LPS (endotoxin 5.5 mg/kg ip), (3) Simvastatin 20 mg/kg (per os) + LPS (non-lethal dose of endotoxin 5.5 mg/kg ip), and (4) Simvastatin 40 mg/kg (per os) + LPS (endotoxin 5.5 mg/kg ip). Simvastatin was given orally via oral gavage in the short pretreatment of 5 days, and LPS at the single dose was administered 1.5 h after the last dose of simvastatin. The animals in the LPS group received the same volume (1 ml/kg) of 0.5% methylcellulose for 5 days, as a vehicle, before LPS injection. Control group received an identical volume of vehicle, without simvastatin or LPS. After LPS administration, the animals were observed continuously for 48 h.

### Histopathological Examination

Forty-eight hours after last treatment, all animals were anesthetized (sodium pentobarbital 30 mg/kg ip, Sigma-Aldrich, St. Louis, MO, United States) and sacrificed. Dissected tissue samples were fixed with 10% neutral formalin solution up to 7 days. Issues were divided into four portions in order to be prepared for making sections. Then, tissue slices were dehydrated in alcohol series, xylol and embedded in paraffin blocks. At least, the staining of paraffin cuts (thickeness 2-μm) was done using haematoxylin and eosin (H&E) method.

### Semiquantitative Analysis

The intensity of hepatic and splenic lesions consisting of oedema, amount of inflammatory cells, hemorrhages, degeneration and their distribution were counted in six separate visual fields at 400× magnification. Degenerative and vascular alterations were analyzed throughout whole visual fields by using a light microscope according to the 5-point semiquantitative scale published earlier in the literature (Jaćević et al., 2016, 2017, 2018; Nežić et al., 2018). A severity grade, shown as hepatic damage score (HDS) and splenic damage score (SDS) of tissue lesions were determined for all sections of the whole tissue, and a mean hepatic and splenic were HDS and SDS determined, respectively. The exact method of calculation is shown in Table 1, respectively.

**Table 1:** Effects of simvastatin on the hepatic damage score (HDS) and splenic damage score (SDS) in LPS-induced hepatic and splenic injury in rats.

Treatment (mg/kg)	0	1	2	3	4	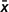 ± S.D.
**(A) Hepatic damages score (HDS) (6 livers/group × 6 slices/liver)**						
Control	36	0	0	0	0	0.00 ± 0.00
LPS 0.25 LD_50_/kg	0	0	0	13	23	3.67 ± 0.55 a^3^
Simvastatin 20 + LPS	0	12	14	10	0	2.00 ± 0.89 a^1^ b^2^
Simvastatin 40 + LPS	0	15	21	0	0	1.50 ± 0.55 b^2^
**(B) Splenic damages score (SDS) (6 spleens/group × 6 slices/spleen)**						
Control	36	0	0	0	0	0.00 ± 0.00
LPS 0.25 LD_50_/kg	0	0	0	9	27	3.75 ± 0.44 a^3^
Simvastatin 20 + LPS	0	6	18	12	0	2.17 ± 0.70 a^1^ b^2^
Simvastatin 40 + LPS	0	24	12	0	0	1.33 ± 0.48 b^2^

*Statistical analysis was performed using the Kruskal–Wallis test. a^*1*^, a^*3*^ - *p* < 0.01, 0.001 in comparison to the control group, b^*2*^ - *p* < 0.001 in comparison to the LPS-only treated group*.

### TUNEL Method – *in situ* Determination of Apoptosis in Rat Hepatic and Splenic Tissue

Apoptosis at a cellular level was assessed by TUNEL (Terminal deoxynucleotidyltransferase-mediated dUTP Nick End Labeling) method. An In Situ Cell Death Detection Kit POD (Roche Molecular Biochemicals, Basel, Switzerland, Cat. No 11 684 817 910) was used to perform TUNEL staining on paraffin-embedded sections of 4–6 μm thickness according to the manufacturer’s instructions. Tissue sections were incubated with anti-fluorescein antibody conjugated with horseradish peroxidase (POD), and then color development was performed using diaminobenzidine (DAB) substrate. Negative (incubation with Label Solution, instead of TUNEL reaction) and positive controls (incubation with DNase I recombinant, grade I) were performed per the manufacturer’s instructions. Two different pathologists assessed Immuno-labeled (TUNEL positive) cells in blinded manner. Quantification of TUNEL positive cells was not performed in necrotic areas. The slides were examined under a microscope (Olympus Plaza, Tokyo, Japan) at 400× magnifications. Ten non-successive fields per sample were counted for the number of TUNEL-positive cells. Apoptotic index (AI) defined as the percentage (%) of apoptotic cells was calculated according to the formula: AI (% of apoptotic cells) = The number of TUNEL-positive cells × 100/Total number of cells.

### Immunohistochemical Analysis of Apoptotic Cell Death-Regulating Molecules

Liver and spleen paraffin-embedded sections were stained with polyclonal rabbit antibodies for pro-apoptotic cleaved caspase-3 (Asp 175) (9661, Cell Signaling Technology, Frankfurt, Germany), and anti-apoptotic Bcl-XL, member of the Bcl-2 family of proteins (PA1-37160, Scientific Pierce Product, Rockford, IL, United States), survivin monoclonal mouse antibodies for survivin clone 8E2 (MS-1201-P1 NeoMarkers Inc., Fremont, CA, United States), and NF κB/p65 (RB-1638-R7 Neo-Markers Inc., Fremont, CA, United States), according to the manufacturer’s instructions.

Immunohistochemical analysis was performed on 34 μm deparafinated and rehydrated tissue sections. Slides were then boiled for 20 min in a microwave oven with a citric acid buffer solution (0.01 mol/L citrate buffer, pH 6.0.). To reduce nonspecific background staining slides were incubated in 3% hydrogen peroxide for 10 min. Primary antibodies for cleaved caspase-3 (1:300), Bcl-XL (RTU), survivin (1:50) and NF-κB/p65 (RTU) were applied according to manufacturer’s recommended protocol. The slides were washed thoroughly with phosphate buffered saline, pH 7.4 between the steps. 3,3’-Diaminobenzidine (DAB) (TL-015-HDJ, Thermo Scientific Lab Vision UltraVision ONE Detection System) was used as chromogen, to develop the antigen-antibody complex, and all slides were then counterstained with H&E, dehydrated, and mounted.

In parallel, appropriate positive and negative controls were processed. The slides were analyzed with a microscope (Olympus, Tokyo, Japan) at 400× magnification. Ten non-successive fields per sample were counted for the number of immune-positive cells (cells with expression of cleaved caspase-3, BCL-XL, survivin, and NF-κB/p65), by two independent pathologists in a blinded manner, using ImageJ software (National Institute of Health, Bethesda, MD, United States). Survivin expression were evaluated qualitatively (positive or negative) and considered positive in the presence of cytoplasmic staining in hepatocytes and lymphocytes (Wang et al., 2010; Scheer et al., 2017). Although cell cytoplasmic expression of NF-κB/p65 is detected in most normal cells, only brown nuclear immunostaining is considered as activated NF-κB/p65 and was quantified (Marconi et al., 2014; Nežić et al., 2018). The number of immunopositive hepatocytes and lymphocytes (for survivin and NF-κB/p65, respectively) was expressed as % of positively stained cells = The number of positively stained cells × 100/Total number of cells.

### Statistical Analysis

Data were statistically analyzed by the statistical software package SPSS 19.0. (IBM Corporation, New York, NY, United States). In case of continuous data, variables were presented as mean value ± standard deviation (S.D.). To test the differences in TDS between groups we used the Kruskal–Wallis rank test. Analysis of variance (ANOVA) followed by the Tamhane’s T2 *post hoc* test were used to compare the differences in biomarkers expression among groups. Correlation analysis was performed with Pearson’s correlation coefficient. A value *p* < 0.05 was considered statistically significant.

## Results

### Protective Effects of Simvastatin on Histopathology of Hepatic Tissue in the LPS-Induced Injury

**Figure 1:**
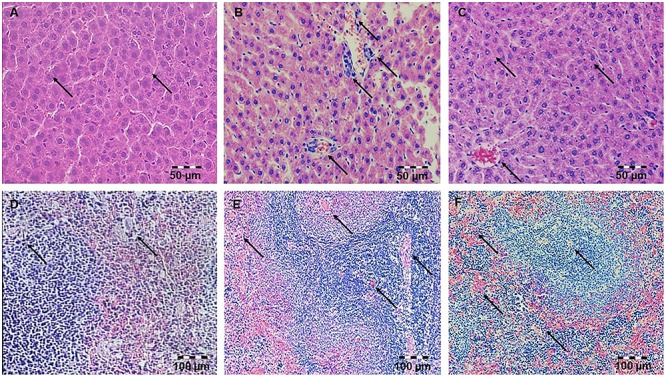
Pretreatment with simvastatin protected from LPS-induced hepatic and splenic injury. Light micrographs of the hepatic tissue, H&E, magnifications 200×, **(A)** Control group. **(B)** LPS group. **(C)** Simvastatin 40 mg/kg group + LPS. Note decreased inflammatory injury and maintenance of the normal histology of the simvastatin-treated liver in contrast to the deterioration of hepatic tissue in the LPS-challenged group. Light micrographs of the splenic tissue, H&E, magnifications 200×, **(D)** Control group. **(E)** LPS group. **(F)** Simvastatin 40 mg/kg group + LPS. Note decreased inflammatory injury and maintenance of the normal histology of the simvastatin-treated spleen in contrast to the deterioration of splenic tissue in the LPS-challenged group.

Hepatic tissue of the control rats has normal histological structure (Figure 1A). Treatment with LPS moderate to severe degenerative changes, and the presence of polymorphonuclear granulocytes, lymphocytes, macrophages and eosinophilic leukocytes (Figure 1B). Such, average HDS was 3.67 ± 0.55. This value was statistically different from the unprotected group (Table 1A). Small necrotic foci and the presence of neutrophils were seen in hepatic tissue of rats protected with simvastatin 20 mg/kg (HDS = 2.00 ± 0.89, *p* < 0.01 vs. LPS group). These alterations were not found only in a group of rats treated with simvastatin 40 mg/kg (Figure 1C). Semiquantitative analysis of these lesions demonstrated the lowest scores for hepatic injuries (HDS = 1.50 ± 0.55) when compared to LPS-treated rats only (*p* < 0.01).

### Protective Effects of Simvastatin on Histopathology of Splenic Tissue in the LPS-Induced Injury

Splenic tissue of the control animals has normal histological structure (Figure 1D). In the LPS-treated group splenic tissue damage ranged from, diffuse sinusoidal oedema, hyperemia and hemosiderosis. The lymphatic tissue was atrophic with a reduced number of lymphocytes, plasmocytes, and macrophages. In addition, a greater number of degenerative lymphocytes and the presence of necrosis in follicles of white pulp were observed (Figure 1E).

The intensity of LPS-induced splenic injury was designated as severe with SDS = 3.75 ± 0.44 (Table 1B), whereas pre-treatment with simvastatin significantly decreased injury induced by LPS (Figure 1F).

As Figure 1 shows, in the simvastatin 40 mg/kg group’s near-normal histological structure, but oedema and hyperemia of trabeculae and red pulp of the spleen are significantly reduced and remained focally. The lymphoreticular tissue of white pulp was mildly atrophic, with normal lymphocytes, plasmocytes and macrophages. In addition, individual lymphocytes were degenerated, which could result in lower immunosuppression (Figure 1F). A mean SDS was minimal compare to the control rats (*p* < 0.05) and LPS group (*p* < 0.01), respectively. Splenic structure in the simvastatin 40 group is mainly unaffected (SDS = 1.33 ± 0.48, *p* < 0.01), indicating that simvastatin dose-dependently ameliorated LPS-induced splenic tissue injury.

### Simvastatin Attenuated LPS-Induced Cleaved Caspase-3 Expression and Apoptotic Cell Death in Hepatocytes and Lymphocytes

**Figure 2:**
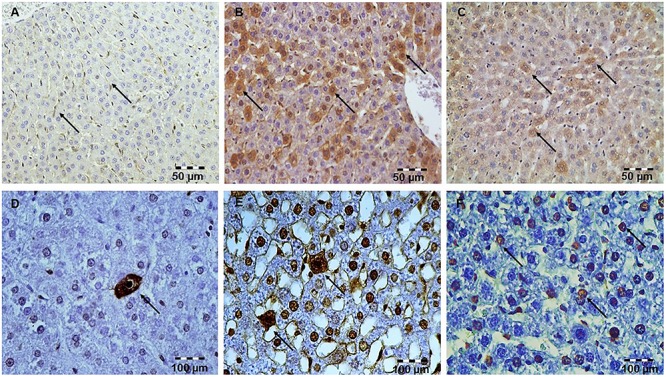
Simvastatin 20 or 40 mg/kg pretreatment attenuated LPS-induced apoptosis throughout the liver by down-regulating cleaved caspase-3 expression in hepatocytes. Magnification 400×. **(A)** Control group. **(B)** Intense cytoplasmic staining of cleaved caspase 3 in hepatocytes LPS group, as characteristic of cell in apoptosis. **(C)** Marked reduction of apoptotic cells in the groups pretreated with Simvastatin 40 mg/kg. Nuclei that were stained dark brown were considered apoptotic (positive) following TUNEL stain. Magnification 400×. The extent of hepatocytes apoptosis increased significantly in the LPS group **(E)** compared with rare TUNEL-positive cells in the control group **(D)** and significantly reduced with Simvastatin 40 mg/kg **(F)**.

Our previous work (Nežić et al., 2009b) confirmed that 10 mg/kg simvastatin had no significant anti-inflammatory effects and survival rate against LPS, and therefore we analyzed apoptosis in the tissue sections challenged with the doses of 20 and 40 mg/kg. Analysis of the extent of apoptosis was performed by the immunohistochemically-determined expression of the main executioner of apoptosis cleaved caspase-3 and TUNEL assay with quantification of apoptotic cells (Figure 2A–F, 3A–F). LPS induced cleavage (activation) of caspase-3 throughout the liver parenchyma and in the white pulp of spleen, characteristically localized in the cytoplasm and perinuclear region of apoptotic cells.

**Figure 3:**
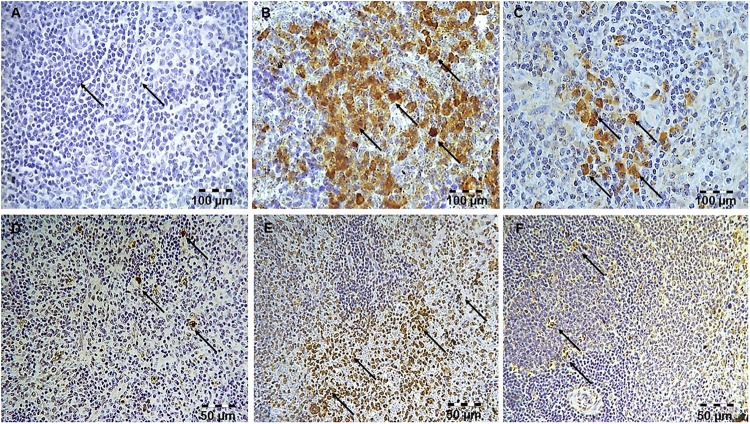
Simvastatin attenuated LPS-induced apoptosis in the spleen by down-regulating cleaved caspase 3 expressions. Magnification 200x. **(A)** Control group. **(B)** Intense brown staining of cleaved caspase 3 in lymphocytes LPS group, as characteristic of cell in apoptosis. Marked reduction of apoptotic cells in the groups pretreated with Simvastatin 40 mg/kg **(C)**. Simvastatin inhibited LPS-apoptosis in the spleen detected by TUNEL staining. Nuclei that were stained dark brown were considered apoptotic following TUNEL stain, magnification 200×. The extent of spleen lymphocytes apoptosis increased significantly in the LPS group **(E)** compared with rare TUNEL-positive cells in the control group **(D)**, and significantly reduced in the groups pretreated with Simvastatin 40 mg/kg **(F)**.

**Figure 4:**
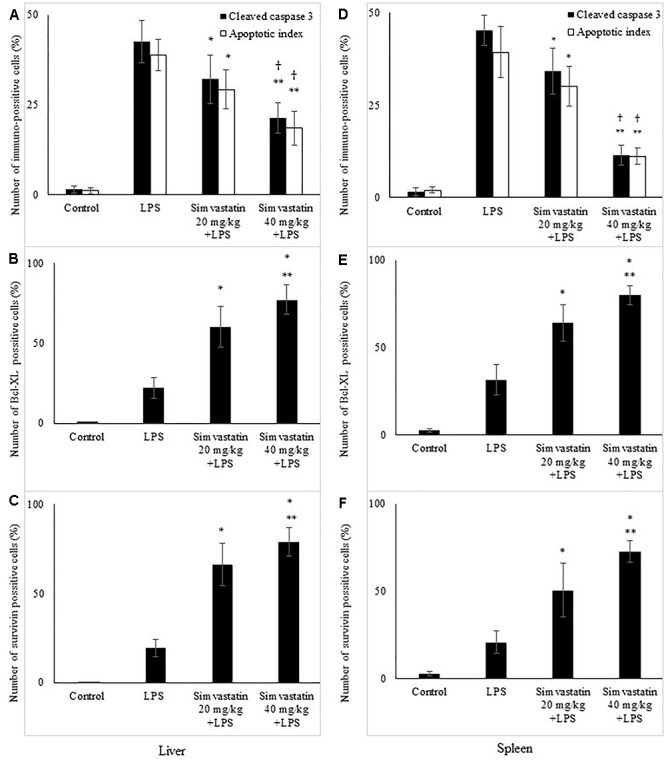
Quantitative analysis is shown for apoptotic cells assessed in immunohistochemically stained sections in liver and spleen. Liver, **(A)** Cleaved caspase 3 and corresponding frequencies of TUNEL positive cells expressed as AI (^∗^*p <* 0.05 vs. LPS group, ^∗∗^*p <* 0.01 vs. LPS group, ^†^*p <* 0.05 vs. simvastatin 20 mg/kg + LPS group), **(B)** Bcl-XL expression in hepatocytes in the selected fields (^∗^*p* < 0.01 vs. LPS group, ^∗∗^*p* < 0.05 vs. simvastatin 20 mg/kg + LPS group), **(C)** Survivin expression (^∗^*p* < 0.01 vs. LPS group, ^∗∗^*p* < 0.05 vs. simvastatin 20 mg/kg + LPS group). Spleen, **(D)** Cleaved caspase 3 and corresponding frequencies of TUNEL positive cells expressed as AI (^∗^*p <* 0.05 vs. LPS group, ^∗∗^*p* < 0.01 vs. LPS group, ^†^*p* < 0.05 vs. simvastatin 20 mg/kg + LPS group), **(E)** Bcl-XL expression in lymphocytes in the selected fields (^∗^*p* < 0.01 vs. LPS group, ^∗∗^*p* < 0.05 vs. simvastatin 20 mg/kg + LPS group), **(F)** Survivin expression (^∗^*p* < 0.01 vs. LPS group, ^∗∗^*p* < 0.01 vs. simvastatin 20 mg/kg + LPS group).

The marked increase of cleaved caspase-3 expression in hepatocytes and lymphocytes induced by LPS (42.6 ± 7.5 and 45.2 ± 5.1%, *p* < 0.001 vs. control group, respectively), was dose-dependently reduced with simvastatin pre-treatment so that 40 mg/kg demonstrated the most profound decline (liver 21.4 ± 5.5%, *p* < 0.05, and spleen 11.8 ± 3.1%, *p* < 0.01 vs. simvastatin 20 mg/kg), and highly significant in comparison to the LPS group (*p* < 0.01) (Figure 4A,D). The TUNEL assay was used to detect DNA fragmentation characteristic for apoptosis, seen as nuclei stained dark brown. In control sections, TUNEL positive cells were sporadically found. A number of TUNEL positive cells substantially increased after LPS challenge in the liver (AI = 38.56 ± 8.5%) and spleen (AI = 40.2 ± 10.1%). However, pre-treatment with simvastatin demonstrated dose-dependent decline of hepatocytes and splenic lymphocytes apoptosis, with the most intense effect of simvastatin 40 mg/kg in the liver (AI = 18.2 ± 3.6%, Figure 2F, 4A) and spleen (11.2 ± 2.4%, Figure 3F, 4D) as compared with LPS group (*p* < 0.01).

Expression of cleaved caspase-3 indicates apoptotic but also pre-apoptotic cells without chromatin condensation and change of cell morphology. Although we determined non-significant differences in the total number of immune-positive cleaved caspase-3 cells and AIs determined by TUNEL assay, a very strong positive correlation was found between them across the groups (*p* < 0.05).

### Simvastatin Enhanced Expression of Anti-apoptotic Bcl-xL in Hepatocytes and Lymphocytes in LPS-Induced Inflammation

**Figure 5:**
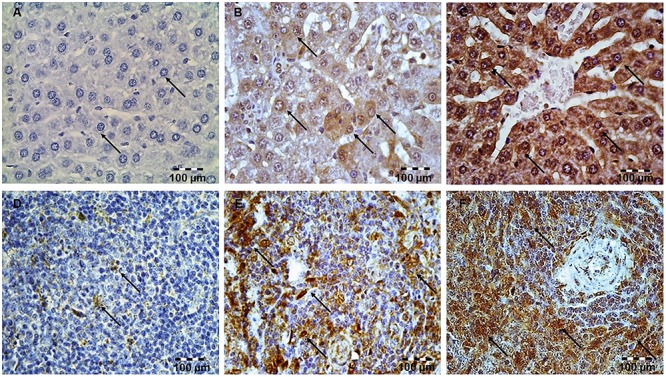
Simvastatin increased Bcl-XL expression in rat liver in LPS-induced inflammation. Distribution of Bcl-XL-positive hepatocytes that were challenged with a non-lethal dose of LPS or either pretreated with simvastatin 40 mg/kg before LPS, magnification 400×. **(A)** Control group. **(B)** Note focally distributed BCL-XL-positive hepatocytes in the LPS group, **(C)** Simvastatin 40 mg/kg + LPS group. Simvastatin increased Bcl-XL expression in rat spleen in LPS-induced inflammation. Distribution of Bcl-XL-positive lymphocytes that were challenged with a non-lethal dose of LPS or either pretreated with simvastatin 40 mg/kg before LPS, magnification 400×. **(D)** Control group. **(E)** Note a significant number of BCL-XL-positive lymphocytes in the LPS group, and **(F)** Simvastatin 40 mg/kg + LPS group.

Immunohistochemically staining of Bcl-xL in hepatocytes and lymphocytes of white pulp showed a marked difference in the expression among the LPS and simvastatin groups. Results showed weak and rare Bcl-XL expression in the control only in the white pulp and in contrast a significant increase of immune-positive hepatocytes and lymphocytes the LPS groups (*p* < 0.05 vs. control group) (Figure 5A–F). Determined expression of Bcl-xL following LPS challenge might represent induction of the cell self-protective mechanism in the internal apoptotic pathway. However, the extensive Bcl-XL expression was determined in the simvastatin groups in the positive cells showing brown intense diffuse cytoplasmic staining throughout the parenchyma. Pre-treatment with simvastatin 40 mg/kg produced the most pronounced increase of Bcl-XL-positive hepatocytes and lymphocytes (77.4 ± 9.3% and 80.2 ± 4.7%, respectively) compared to simvastatin 20 mg/kg (*p* < 0.05), and to LPS group (*p* < 0.01) (Figure 4B,E). These results indicate that simvastatin might have cell protective properties through induction of anti-apoptotic Bcl-XL.

### Simvastatin Enhanced Survivin Expression in Hepatocytes and Lymphocytes in LPS-Induced Inflammation

**Figure 6:**
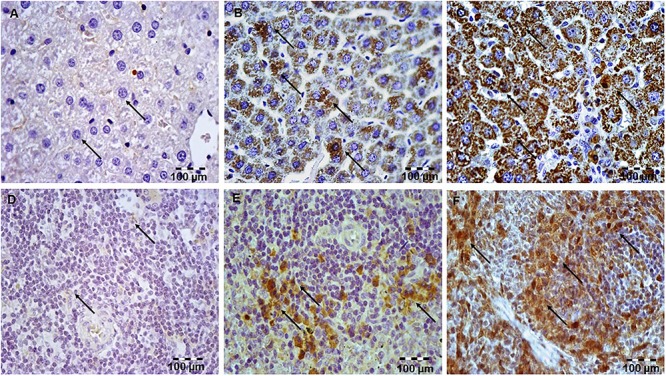
Simvastatin increased survivin expression in rat liver in LPS-induced inflammation. Distribution of survivin-positive hepatocytes that were challenged with a non-lethal dose of LPS or either pretreated with simvastatin 40 mg/kg before LPS, magnification 400x. **(A)** Control group. **(B)** Note significant increase in the number of survivin-positive hepatocytes with intensive cytoplasmic staining in the LPS group, **(C)** Simvastatin 40 mg/kg + LPS group. Simvastatin increased survivin expression in rat spleen in LPS-induced inflammation. Distribution of survivin-positive lymphocytes that were challenged with a non-lethal dose of LPS or either pretreated with simvastatin 40 mg/kg before LPS, magnification 400×. **(D)** Control group. **(E)** Note significant increase in a number of survivin-positive lymphocytes with intensive cytoplasmic staining in the LPS group, and **(F)** Simvastatin 40 mg/kg + LPS group.

Having demonstrated that simvastatin enhanced Bcl-XL expression in LPS-induced inflammation, we further determined its anti-apoptotic mechanism in the hepatocytes and lymphocytes. In the control group, survivin expression was rarely detected in the white pulp. As illustrated in Figure 6, LPS challenge resulted in a marked increase of survivin expression, suggesting that survivin itself plays a role in cell-protective mechanism in acute LPS injury (*p* < 0.05 vs. control group). More importantly, simvastatin, dose-dependently enhanced survivin expression in the parenchyma as demonstrated as a strong cytoplasmic staining in hepatocytes and lymphocytes (*p* < 0.01 vs. LPS group) (Figure 4C,F, 6).

**Figure 7:**
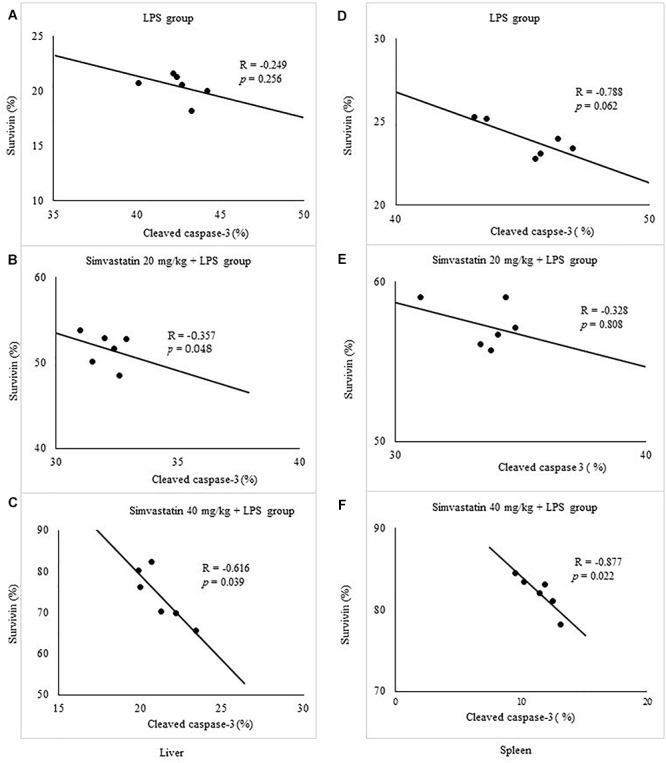
Correlations are shown for apoptotic cells assessed in immunohistochemically stained liver and spleen sections for cleaved caspase 3 and survivin expression in hepatocytes and lymphocytes. Liver, **(A)** LPS group, **(B)** Simvastatin 20 mg/kg + LPS group, **(C)** Simvastatin 40 mg/kg + LPS group. Spleen, **(D)** LPS group, **(E)** Simvastatin 20 mg/kg + LPS group, **(F)** Simvastatin 40 mg/kg + LPS group.

Quantitative analysis of the immune-positive cell revealed that simvastatin 40 mg/kg led to a dramatic increase in survivin expression in the hepatocytes distributed throughout the liver parenchyma (79.1 ± 14.2%) and lymphocytes of germinal centers (72.8 ± 6.7%) (Figure 7C,F). As survivin is one of IAPs, we further analyzed its correlation with a key marker of apoptosis in selected tissue sections (*n* = 12). As illustrated in Figure 7A–C, a significant moderate to strong negative correlations were found between survivin and cleaved caspase-3 in simvastatin 20 and 40 mg/kg (*R* = -0.357; *p* = 0.048 and *R* = -0616; *p* = 0.039, respectively) in hepatocytes, as well as in lymphocytes in simvastatin 40 mg/kg group (*R* = -0.877, *p* = 0,022), suggesting that simvastatin enhances cell survival against LPS (Figure 7D–F).

### Simvastatin Increased Expression of NF-κB/p65 in Hepatocytes and Lymphocytes in LPS-Induced Inflammation

To obtain more evidence that simvastatin enhances cell survival against LPS, we analyzed the NF-κB expression profile as one of the main transcriptional factors that up-regulate survivin.

**Figure 8:**
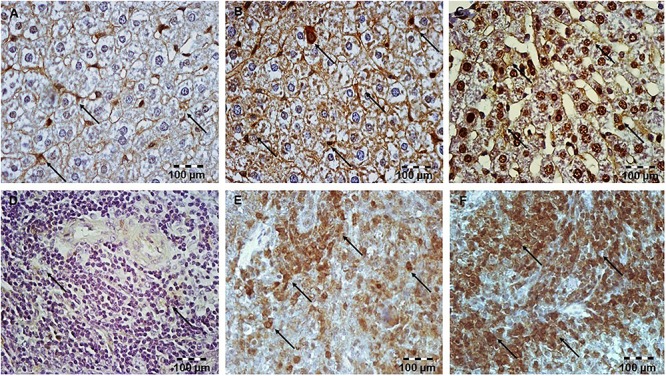
Simvastatin increased NF-κB expression in rat liver tissue in LPS-induced inflammation. Distribution of NF-κB-positive hepatocytes that were challenged with a non-lethal dose of LPS or either pretreated with simvastatin 40 mg/kg before LPS, magnification 400×. **(A)** Control group. Note focal hepatocytes positive for NF-κB/p65 (nuclear staining) in the LPS group **(B)**, and a significant increase in NF-κB-positive hepatocytes **(C)** Simvastatin 40 mg/kg + LPS group. Simvastatin increased NF-κB expression in rat spleen tissue in LPS-induced inflammation. Distribution of NF-κB-positive lymphocytes that were challenged with a non-lethal dose of LPS or either pretreated with simvastatin 20 mg/kg or simvastatin 40 mg/kg before LPS, magnification 400×. **(D)** Control group. **(E)** Note a significant number of lymphocytes positive for NF-κB/p65 (nuclear staining) in the LPS group, and the strongest increase in NF-κB-positive lymphocytes, and **(F)** Simvastatin 40 mg/kg + LPS group.

Immunohistochemical staining shows activation of NF-κB as nuclear staining, after it is as translocation from the cytoplasm to the nucleus. Upon LPS administration, focally distributed rare hepatocytes and a subset of lymphocytes were positive for NF-κB in the cell cytoplasm and/or nucleus (Figure 8A–C). As shown in Figure 8D–F in lymphocytes of the control group, positive immune reaction for NF-κB was undetectable, while weak cytoplasmic expression in rare hepatocytes was considered as its activity in basal condition. Furthermore, simvastatin groups demonstrated a significant increase in positive NF-κB hepatocytes and lymphocytes, compared to the LPS group (*p* < 0.01).

**Figure 9:**
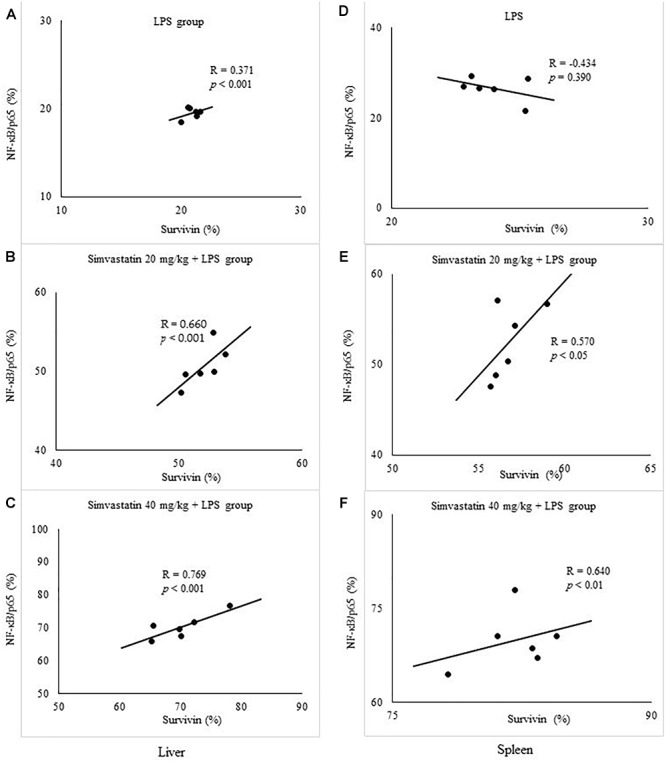
Correlation is shown for apoptotic cells assessed in immunohistochemically stained liver and spleen sections for survivin and NF-κB/p65 expression in hepatocytes and lymphocytes. Liver, **(A)** LPS group. **(B)** Simvastatin 20 mg/kg + LPS group. **(C)** Simvastatin 40 mg/kg + LPS group. Spleen, **(D)** LPS group. **(E)** Simvastatin 20 mg/kg + LPS group. **(F)** Simvastatin 40 mg/kg + LPS group.

To further confirm the expression pattern and correlation of NF-κB and survivin in LPS-induced inflammation we tested the correlation between their expressions in the twelve selected liver and spleen sections in the experimental groups. Consistently, Pearson’s correlation analysis suggested a significant strong positive correlation with NF-κB positive hepatocytes in simvastatin 20 mg/kg (*R* = 0.66, *p* < 0.001), and 40 mg/kg group (*R* = 0.76, *p* < 0.001). Similarly, in the lymphocytes, survivin-positive cells also demonstrated a significant strong correlation with NF-κB activation in simvastatin 20 and 40 mg/kg group (*R* = 0.57, *p* < 0.05, and *R* = 0.64, *p* < 0.01, respectively). Taken together, these results suggest that up-regulation of survivin is associated with NF-κB activation that is strongly enhanced by simvastatin (Figure 9A–C, D–F).

## Discussion

In this study, several major observations relevant to protection of LPS-induced organ injuries using LPS experimental model can be emphasized: (1) simvastatin, in a dose-dependent manner, attenuated liver, and spleen inflammatory injuries along to reduced extent of LPS-induced apoptotic-cell death in the parenchyma; (2) LPS led to increased protein expression of anti-apoptotic molecules Bcl-XL and survivin, in hepatocytes and lymphocytes, that most probably serve as an induction of cell-protection mechanism; (3) simvastatin significantly increased cytoplasmic expression of survivin that is associated with marked activation of NF-κB/p65, indicating that NF-κB/p6/survivin signaling can be mechanism of simvastatin cell protective effects in experimental sepsis.

Direct evidence in favor of pre-treatment with statins can be derived from numerous experimental studies, which demonstrated that statins improved survival, organ functions, and prevent organ injuries in rat/murine model of sepsis with LPS or cecal ligation puncture (CLP) (Merx et al., 2004; Shinozaki et al., 2010; Slotta et al., 2010; Wang et al., 2018). Herein, we demonstrated that simvastatin pretreatment attenuated liver and spleen injuries against LPS, seen as significantly reduced inflammatory infiltrates associated with minimal alterations in histological structure. These findings are supported by our and other’s previous results that orally given simvastatin dose-dependently decreased TNF-α and IL-1β (Nežić et al., 2009a,b), that inhibit leukocyte recruitment and adhesion stimulated by TNF-α via interference with both adhesion molecule (P-selectin) expression and function (LFA-1) (Slotta et al., 2010).

It has been purposed that hepatocytes apoptosis is an important mechanism in LPS induced liver injury (Shinozaki et al., 2010; La Mura et al., 2013; Wang et al., 2018). Herewith, the significant apoptosis of lymphocytes might be an underlying cause of the profound immunosuppression that is a critical point in patients with sepsis (Hotchkiss et al., 2013). In the present study, we observed that simvastatin abolished LPS-induced cleavage of caspase-3 in hepatocytes and splenic lymphocytes and apoptotic cell death, and markedly increased Bcl-XL expression, supporting data of cell-protective effects of statins against endotoxemic organ injuries. Experimental studies also confirmed that simvastatin reduced LPS-induced hepatocytes apoptosis in partial hepatectomy (Slotta et al., 2010), decreased caspase-3 expression (Shinozaki et al., 2010), and inhibited TNF-α/caspase-3 pathway in burn injuries (Zhao et al., 2013). According to our results, simvastatin (Zhang et al., 2012; Zhao et al., 2015) similarly as ulinastatin, a protease inhibitor (Huang et al., 2013), attenuated splenic T-cell apoptosis and increased anti-apoptotic Bcl-2 expression in abdominal (CLP) sepsis and burn injury. Results also confirmed that simvastatin similar as ulinastatin, inhibits TNF-α, an extrinsic trigger of apoptosis that leads to activation of caspase-3 (Nežić et al., 2009b; Slotta et al., 2010; Huang et al., 2013; Zhao et al., 2013, 2015).

It has been well documented that statins inhibithydroxy-3-methylglutaryl-CoA reductase and block mevalonate pathway that leads to reduced protein isoprenylation, namely farnesylation and geranylgeranylation, and prevents activation of RAS protein, key signaling molecules involved in the regulation of apoptosis, survival, proliferation, and differentiation. In accordance with these findings, previous study showed that simvastatin and farnesyltransferase inhibitor decrease apoptosis (inhibits caspase-3, Bax and Bim expression) of hepatocytes and spleen lymphocytes in endotoxemia (Shinozaki et al., 2010). This might partly explain anti-apoptotic effects of simvastatin, but its exact mechanism in inhibiting apoptosis in sepsis needs further investigation.

Survivin is the smallest IAPs, which is expressed in several apoptosis-regulated fetal tissues, whereas in adults non-malignant conditions are indispensable for homeostasis of the immune system, such as innate and adaptive immune response such as lymphocytes’ homeostasis (Gravina et al., 2017). Despite the facts that survivin has been considered as an important signaling molecule in the pathogenesis of numerous autoimmune inflammatory diseases, or its increased expression is confirmed in leukocytes infiltrating ulcerative colitis and lung tissue in cystic fibrosis (Gravina et al., 2017), a potential role of survivin in hepatocytes and lymphocytes survival in sepsis still remains unclear. Our results have demonstrated marked cytoplasmic survivin expression in hepatocytes and lymphocytes after LPS, and we assumed that it presents induction of cell-protection mechanism in the LPS-induced tissue injury. Further, simvastatin-induced intense cytoplasmic expression of survivin in hepatocytes throughout the parenchyma and in the most of lymphocytes in the white pulp that is inversely correlated with apoptotic markers cleaved caspase-3 and TUNEL findings, respectively. This observation indicates that anti-apoptotic effects of simvastatin in LPS-induced inflammatory injury might be partly survivin-dependent. Recent similar results demonstrated that reduced apoptosis of cardiomyocytes in a CLP model sepsis is associated with upregulated survivin and increased expression of thioredoxin-1, an antioxidant cytosolic protein (Wilson et al., 2017). However, not all IAP directly bind to caspase-3 and inhibit apoptosis. Some IAPs, such as survivin activate NF-κB/p65 or induce its activation by TNF-α/TNF receptor interaction (Wang et al., 2010; Hotchkiss et al., 2013). The function of NF-κB is primarily regulated by IκB family members, which ensure NF-κB inactive status in cytoplasm. Upon stimulus-induced IκB degradation, the NF-κB complexes move to the nucleus and activate NF-κB-dependent transcription (Wang, 2015). It has been shown that survivin over expression affects cell apoptosis in esophageal squamous cell carcinoma cells, and activates translocation NF-κBp65 to the nucleus via maintaining a high expression level of inhibitor of nuclear factor κB kinase subunit β (IKKβ) and upregulating the phosphorylation level of IκBα via IKKβ (Zeng et al., 2016). On the other hand, Cui et al. (2017) provided evidence that survivin is positioned at the downstream in NF-κB signaling pathway a in bladder cancer progression, NF-κB activation enhances the expression of survivin both *in vitro* and *in vivo*. At present, we partially understood the correlation of NF-κB and survivin in apoptosis in inflammatory tissue injury.

Therefore, we next asked whether simvastatin might influence NF-κB activation, which could be associated with survivin upregulation in intracellular survival pathway. Indeed, we observed that significant nuclear NF-κB/p65 expression, in hepatocytes and splenic lymphocytes, is enhanced by simvastatin, and additionally in strong positive correlation with survivin expression. Therefore, based on these results we assume that survivin/NF-κB/p65 pathway is activated in LPS-induced inflammatory injury, and that simvastatin through this pathway can improve cell survival. Our recent study supports this as we showed that simvastatin-induced anti-apoptotic Bcl-Xl and survivin/NF-κB/p65 expression in cardiomyocytes after LPS exposure (Nežić et al., 2018). Similar activation of NF-κB associated with survivin upregulation was observed in hepatocytes against cytotoxic glycochenodeoxycholate (Wang et al., 2010). Taken together, it should be noted that simvastatin is not only an important inhibitor of apoptotic cell death in septic liver and spleen but also a potent inducer of cell-survival pathways in hepatocytes and lymphocytes.

## Conclusion

This study demonstrated that in LPS induces apoptosis of hepatocytes and lymphocytes, but triggers expression of cell survival markers such as Bcl-XL and survivin, which are significantly enhanced by simvastatin. Significantly, increased expression of survivin associated with NF-κB/p65 activation seems that contribute to the anti-apoptotic effects of simvastatin. These data have broadened our understanding of simvastatin anti-apoptotic and cell-protective effects in experimental LPS-induced inflammatory injury, that may serve as the rationale for the clinical use of statins in prevention of immunosuppression and liver injury in sepsis, but this warrants further clinical investigation.

## Author Contributions

LN, LA, RŠ, and VJ contributed to the conception, design, and preparation of the manuscript. VJ was responsible for the experiments performed in animals, H&E method, and semiquantitative analysis. LA and RG were independently responsible for all immunohistochemical analyses. LN, RŠ, EN, MV, and KK revised the manuscript. LN and VJ were responsible for the final approval of the version to be published. All authors were agreed to be accountable for all aspects of the work.

## Conflict of Interest Statement

The authors declare that the research was conducted in the absence of any commercial or financial relationships that could be construed as a potential conflict of interest.
